# OR11-003 - The NLRP3 inflammasome is regulated by CaSR

**DOI:** 10.1186/1546-0096-11-S1-A192

**Published:** 2013-11-08

**Authors:** JJ Chae, G-S Lee, N Subramanian, DL Kastner

**Affiliations:** 1 Medical Genetics Branch, National Human Genome Research Institute, Bethesda, USA; 2 Laboratory of Systems Biology, National Institute of Allergy and Infectious Diseases, Bethesda, USA

## Introduction

Mutations in the gene encoding NLRP3 cause a spectrum of autoinflammatory diseases known as the cryopyrin-associated periodic syndromes (CAPS). NLRP3 is a key component of one of several distinct cytoplasmic multiprotein complexes (inflammasomes) that mediate the maturation of the proinflammatory cytokine interleukin-1β (IL-1β) by activating caspase-1. Although several models for inflammasome activation, such as K^+^ efflux, generation of reactive oxygen species, and lysosomal destabilization have been proposed, the precise molecular mechanism of NLRP3 inflammasome activation, as well as the mechanism by which CAPS-associated mutations activate NLRP3, remains to be elucidated.

## Objectives

To investigate how extracellular DAMP signals activate the NLRP3 inflammasome and the molecular pathogenesis of CAPS.

## Methods

Using a combination of genetic, pharmacological, and biochemical approaches, we provide evidence that the CaSR is essential for NLRP3 inflammasome activation, which is directly controlled by intracellular Ca^2+^ and cAMP.

## Results

Ca^2+^ or other CaSR agonists activate the NLRP3 inflammasome in the absence of exogenous ATP, whereas knockdown of *CaSR* reduces inflammasome activation in response to known NLRP3 activators. The CaSR activates the NLRP3 inflammasome through phospholipase C (PLC), which catalyzes inositol trisphosphate (IP_3_) production and thereby induces release of Ca^2+^ from endoplasmic reticulum (ER) stores. The increased cytoplasmic Ca^2+^ promotes the assembly of inflammasome components, and intracellular Ca^2+^ is required for spontaneous inflammasome activity in cells from CAPS patients. CaSR stimulation also results in reduced intracellular cAMP, which independently activates the NLRP3 inflammasome. cAMP binds to NLRP3 directly to inhibit inflammasome assembly, and downregulation of cAMP relieves this inhibition. The binding affinity of cAMP for CAPS-associated mutant NLRP3 is substantially lower than for wild-type NLRP3, and the uncontrolled mature IL-1β production from CAPS patients’ peripheral blood mononuclear cells is attenuated by increasing cAMP.

## Conclusion

Taken together, these findings suggest that Ca^2+^ and cAMP are two key molecular regulators of the NLRP3 inflammasome that have critical roles in the molecular pathogenesis of CAPS. In addition, our data suggest a broader spectrum of potential targets for therapy of CAPS as well as other inflammatory conditions involving the NLRP3 inflammasome, including gout, type 2 diabetes mellitus, atherosclerosis, and Alzheimer’s disease.

## Competing interests

None declared.

